# Synthesis of aminophenylhydroxamate and aminobenzylhydroxamate derivatives and *in vitro* screening for antiparasitic and histone deacetylase inhibitory activity

**DOI:** 10.1016/j.ijpddr.2018.01.002

**Published:** 2018-01-31

**Authors:** C. Loeuillet, B. Touquet, B. Oury, N. Eddaikra, J.L. Pons, J.F. Guichou, G. Labesse, D. Sereno

**Affiliations:** a Univ. Grenoble Alpes, CNRS, CHU Grenoble Alpes, Grenoble INP, TIMC-IMAG, F-38000 Grenoble, France; b Institute for Advanced Biosciences (IAB), Team Host-Pathogen Interactions & Immunity to Infection, INSERM U 1209, CNRS UMR 5309, Université Grenoble Alpes, Grenoble, France; c IRD, Univ Montpellier, InterTryp, Montpellier, France; d IRD, Univ Montpellier, MiVegec, Montpellier, France; e Laboratoire d’Eco-épidemiologie Parasitaire et Génétique des Populations, Institut Pasteur d’Alger, Route du Petit Staoueli, Dely Brahim, Alger, Algeria; f Laboratoire de Biochimie Analytique et Biotechnologies, Université Mouloud Mammeri de Tizi Ouzou, Algeria; g Centre de Biochimie Structurale (CBS), INSERM, CNRS, Université de Montpellier, France

## Abstract

A series of aminophenylhydroxamates and aminobenzylhydroxamates were synthesized and screened for their antiparasitic activity against *Leishmania*, *Trypanosoma*, and *Toxoplasma*. Their anti-histone deacetylase (HDAC) potency was determined. Moderate to no antileishmanial or antitrypanosomal activity was found (IC_50_ > 10 μM) that contrast with the highly efficient anti-*Toxoplasma* activity (IC_50_ < 1.0 μM) of these compounds. The antiparasitic activity of the synthetized compounds correlates well with their HDAC inhibitory activity. The best-performing compound (named 363) express a high anti-HDAC6 inhibitory activity (IC_50_ of 0.045 ± 0.015 μM) a moderate cytotoxicity and a high anti-*Toxoplasma* activity in the range of known anti-*Toxoplasma* compounds (IC_50_ of 0.35–2.25 μM). The calculated selectivity index (10–300 using different human cell lines) of the compound 363 makes it a lead compound for the future development of anti-*Toxoplasma* molecules.

## Highlights

• A series of aminophenylhydroxamates and aminobenzylhydroxamates were synthetized.
• They show moderate anti-Trypanososmatids and high anti-*Toxoplasma* activity.
• The *T. gondii* activity correlates with their HDAC inhibitory.
• The best-performing compound had high anti-HDAC6 (class II) activity.
• This compound represents a “lead” for the development of new anti-*Toxoplasma*.

## Graphical abstract

**Figure:**
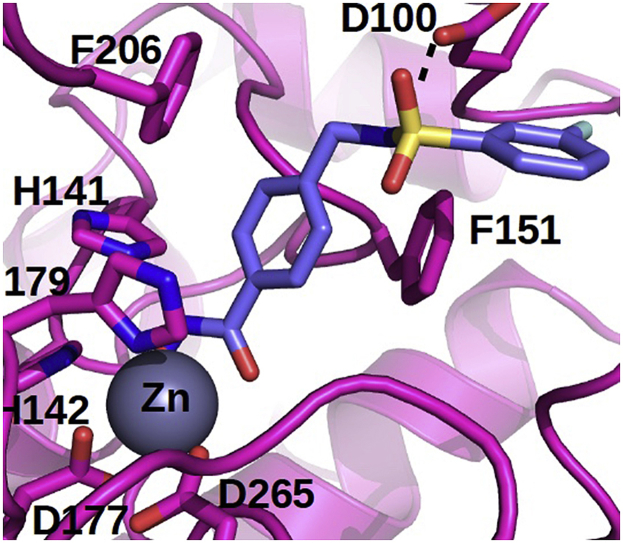

## 1 Introduction

Parasitic diseases cause significant morbidity and mortality infecting hundreds of millions of people particularly in developing countries (Lozano et al., 2012). Human African trypanosomiasis (HAT) or sleeping sickness is caused by two *Trypanosoma brucei* (*T.b.*) subspecies transmitted through the bites of tsetse flies (*Glossina*). Seventy million people risk to contract HAT (Franco et al., 2014). *Trypanosoma cruzi*, the agent of Chagas disease, is mainly transmitted by triatomines, but transmission may occur orally and congenitally, by transfusion or transplantation (Rassi et al., 2010). About 6–7 million people worldwide, mostly in Latin America, are infected. Leishmaniasis is a worldwide threatening problem (Akhoundi et al., 2017). New antileishmanial compounds are currently needed, because of the decreasing efficiency of classical drugs (antimonial containing drugs) reported in various endemic areas (Lira et al., 1999, Sereno et al., 2012, Seblova et al., 2014, Uliana et al., 2016). Altogether, for all these neglected tropical diseases, drugs currently available are few, limited in efficacy display a wide range of side effects and are challenged by drug resistant organisms (see Chellan et al., 2017, Sereno et al., 2012, Stein et al., 2014, Zingales, 2017). All these diseases belong to Neglected tropical Diseases, that affect the poorest individual (Molyneux et al., 2017). *Toxoplasma gondii* is transmitted through meat containing *T. gondii* cysts or through water containing oocysts from feline feces. During the acute phase of infection in immunocompetent patients, the disease is largely asymptomatic but can present as a “flu-like syndrome” in 20% of patients. Severe toxoplasmosis can be observed especially in fetuses and immunocompromised patients, such as transplant and AIDS patients, in whom the infection can lead to encephalitis, chorioretinitis, heart and lung lesions (Tenter et al., 2000).

The discovery of novel drug targets and new chemotherapies with novel mechanisms of action that kill intracellular parasites of humans, are high priorities. Small molecules that act on epigenetic regulatory proteins are of increasing interest as chemical tools for dissecting fundamental mechanisms of parasite growth and as new drug leads. Histone deacetylases (HDACs) play key roles in diverse intracellular processes and epigenetic regulation through the modification of histone and nonhistone proteins to repress transcription. In human cells, 18 HDACs have been identified (Mariadason, 2008) and classified according to their dependency on either zinc or NAD+as the co-factor and to their sequence homology to yeast proteins (Xu et al., 2007). The first evidence of the potential of HDACs as targets for antiparasitic drug development came with the discovery of apicidin, a fungal metabolite that exhibits nanomolar HDAC inhibitor (HDACi) activity and high anti-*Plasmodium falciparum* activity (Darkin-Rattray et al., 1996). Accumulating evidence has indicated that zinc- or NAD-dependent HDACs are promising drug targets in a wide variety of parasitic diseases, including Schistosomiasis, malaria, leishmaniasis, trypanosomiasis and toxoplasmosis (Mai et al., 2004, Saksouk et al., 2005, Sereno et al., 2005, Vergnes et al., 2005, Strobl et al., 2007, Andrews et al., 2012, Soares et al., 2012; Stolfa et al., 2014, Carrillo et al., 2015, Engel et al., 2015, Campo, 2016, Chua et al., 2016) and reviewed by Hailu et al. (2017).

In this study, we synthesized and ascertained the antiparasitic activity, cytotoxicity and HDACi activity of a series of aminophenylhydroxamates and aminobenzylhydroxamates derived from an initial alpha-hydroxyacetone compound (ST3) that have demonstrated an *in vitro* antileishmanial activity associated with a moderate cytoxicity (Sereno et al., 2008).

## 2 Materials and methods

### 2.1 General procedures for the synthesis of aminophenylhydroxamate and aminobenzylhydroxamate derivatives

**Fig. 1:**
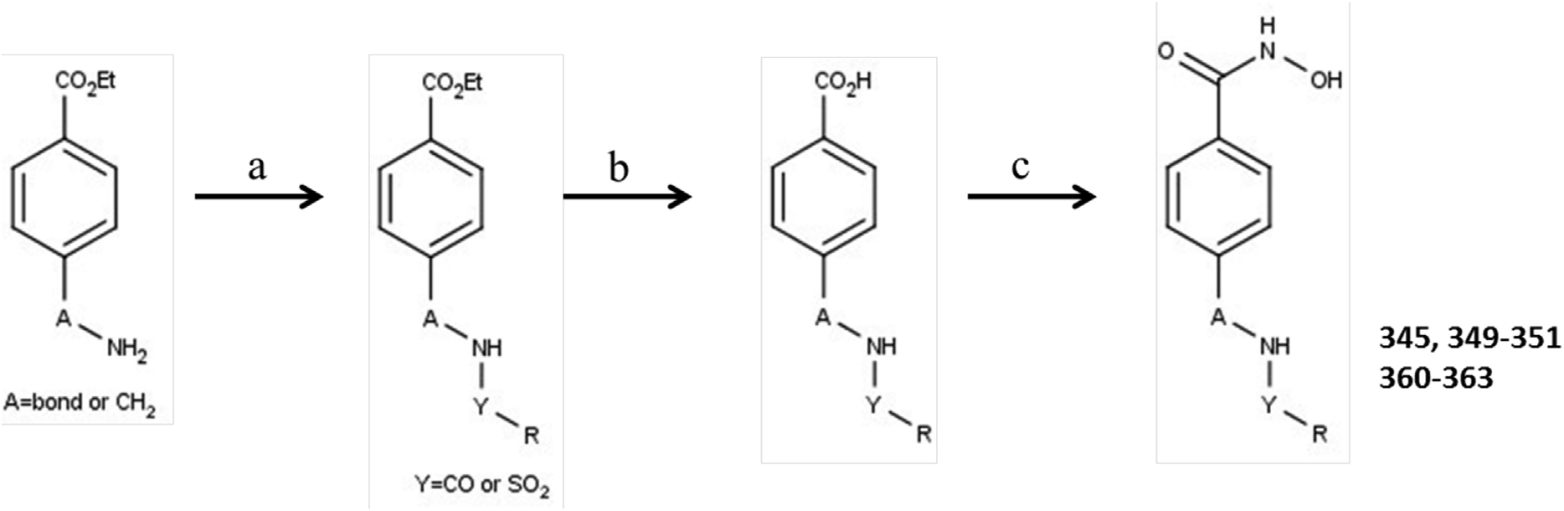
**Synthetic routes of aminophenylhydroxamate and aminobenzylhydroxamate derivatives.** Reagents and conditions: (a) R-Y-Cl, NEt_3_, DCM; (b) LiOH, THF/H_2_O 40 °C; (c) (i) Cl-CO_2_Et, NMM, DMF, (ii) NH_2_OH, MeOH.

A series of aminophenylhydroxamates and aminobenzylhydroxamates were designed from an initial benzohydroxamate compound (ST3) (Sereno et al., 2008, Sereno et al., 2014). Synthesis of compound ST3, aminophenylhydroxamate, and aminobenzylhydroxamate derivatives was performed as previously described (Reddy et al., 2010) and a synthetic view is given in Fig. 1. Amine derivative (1 eq.) was dissolved in dry DCM (0.2 M). Triethylamine (3 eq.) and the acid chloride or sulfonyl chloride (1 eq.) were added successively and the reaction mixture was heated at 40 °C. After the reaction was complete (TLC control), the reaction mixture was concentrated. The residue was taken up with EtOAc and the organic phase was washed with a solution of 1 M HCl, a saturated solution of NaHCO_3_ and brine, dried over Na_2_SO_4_, filtrated and concentrated to afford the amide or sulfonamide derivatives. The products were used such as in the next step. Amide or sulfonamide derivatives (1 eq.) were dissolved in THF (3 ml). A solution of LiOH (3 eq.) in 3 ml of water was added and the reaction mixture was heated at 40 °C overnight. The reaction mixture was concentrated. The residue was taken up with 30 ml of water and the aqueous phase was washed with 20 ml of EtOAc, then the aqueous phase was acidified to pH 2 with a solution of 1 M HCl. The aqueous phase was extracted three times with 20 ml of EtOAc. The organic phases were combined and dried over Na_2_SO_4_, filtrated and concentrated to afford the acid derivatives. The products were used such as in the next step. Acids derivatives (1 eq.) were dissolved in dry DMF (5 ml). Ethyl chloroformate (1.2 eq.) and N-methyl-morpholine (1.3 eq.) were added successively at 0 °C. After 10 mn, a solution of hydroxylamine (2 eq.) in MeOH (10 ml) was added and the reaction mixture was warm up to room temperature and let overnight. The reaction mixture was concentrated. The residue was taken up with EtOAc and the organic phase was washed with a saturated solution of NaHCO_3_ and brine, dried over Na_2_SO_4_, filtrated and concentrated. The crude products were purified by flash chromatography to afford the hydroxamate. The characterization data of compound ST3 was reported in a previous work (Reddy et al., 2010), and the characterization data of compounds 345, 349–351, 360–363 were given a follows and NMR spectra can be found in Supplementary data (S1).

#### 2.1.1 4-{[(3-fluorophenyl)formamido]methyl}-N-hydroxybenzamide (**345**)

^1^H NMR (DMSO-d6) δ 11.17 (s, 1H), 9.19 (t, *J* = 4.0 Hz, 1H), 9.00 (s, 1H), 7.70 (m, 4H), 7.56 (m, 1H), 7.39 (m, 3H), 4.51 (d, *J* = 4.0 Hz, 2H). HPLC purity 99%. MS ESI+H^+^ calc. 289.28 exp. 289.10.

#### 2.1.2 4-(3-fluorobenzamido)-N-hydroxybenzamide (**349**)

^1^H NMR (DMSO-d6) δ 10.40 (s br, 1H), 7.75 (m, 6H), 7.58 (m, 1H), 7.45 (m, 1H).

HPLC purity 98%. MS ESI+H^+^ calc. 275.25 exp. 275.10.

#### 2.1.3 4-(3-fluorobenzenesulfonamidomethyl)-N-hydroxybenzamide (**350**)

^1^H NMR (DMSO-d6) δ 11.17 (s, 1H), 9.02 (s, 1H), 8.37 (s, 1H), 7.60 (m, 4H), 7.50 (m, 2H), 7.29 (d, *J* = 5.6 Hz, 2H), 4.07 (s, 2H). HPLC purity 97%. MS ESI+H^+^ calc. 325.30 exp. 325.10.

#### 2.1.4 4-(3-fluorobenzenesulfonamido)-N-hydroxybenzamide (**351**)

^1^H NMR (DMSO-d6) δ 11.07 (s, 1H), 10.75 (s, 1H), 8.95 (s, 1H), 7.55 (m, 5H), 7.48 (m, 1H), 7.15 (d, *J* = 5.8 Hz, 2H). HPLC purity 99%. MS ESI+H^+^ calc. 311.31 exp. 311.10.

#### 2.1.5 N-{[4-hydroxycarbamoyl)phenyl]methyl}-4-(2-methyl-1,3-thiazol-4-yl)benzamide (**360**)

^1^H NMR (DMSO-d6) δ 11.18 (s, 1H), 9.13 (t, *J* = 4.0 Hz, 1H), 9.01 (s, 1H), 8.09 (s, 1H), 7.98 (d, *J* = Hz, 2H), 7.95 (d, *J* = Hz, 2H), 7.70 (d, *J* = 5.8 Hz, 2H), 7.38 (d, *J* = 5.8 Hz, 2H), 4.52 (d, *J* = 4.0 Hz, 2H), 2.73 (s, 3H). HPLC purity 96%. MS ESI+H^+^ calc. 368.43 exp. 368.10.

#### 2.1.6 N-[4-hydroxycarbamoyl)phenyl]-4-(2-methyl-1,3-thiazol-4-yl)benzamide (**361**)

^1^H NMR (DMSO-d6) δ 11.13 (s, 1H), 10.46 (s, 1H), 8.95 (s, 1H), 8.14 (s, 1H), 8.10 (d, *J* = 4.2 Hz, 2H), 8.03 (d, *J* = 4.2 Hz, 2H), 7.87 (d, *J* = 4.4 Hz, 2H), 7.76 (d, *J* = 4.4 Hz, 2H), 2.75 (s, 3H).

HPLC purity 99%. MS ESI+H^+^ calc. 354.40 exp. 354.10.

#### 2.1.7 N-hydroxy-4-{[2-(3-methoxyphenyl)acetamido]methyl}benzamide (**362**)

^1^H NMR (DMSO-d6) δ 11.17 (s, 1H), 8.99 (s, 1H), 8.58 (t, *J* = 4.0 Hz, 1H), 7.67 (d, *J* = 5.4 Hz, 2H), 7.29 (d, *J* = 5.4 Hz, 2H), 7.19 (t, *J* = 5.8 Hz, 1H), 6.80 (m, 3H), 4.30 (d, *J* = 4.0 Hz, 2H), 3.73 (s, 3H), 3.45 (s, 2H). HPLC purity 99%. MS ESI+H^+^ calc. 315.34 exp. 315.10.

#### 2.1.8 N-hydroxy-4-[2-(3-methoxyphenyl)acetamido]benzamide (**363**)

^1^H NMR (DMSO-d6) δ 11.09 (s, 1H), 10.35 (s, 1H), 8.93 (s, 1H), 7.70 (d, *J* = 5.8 Hz, 2H), 7.64 (d, *J* = 5.8 Hz, 2H), 7.22 (t, *J* = 5.4 Hz, 1H), 6.89 (m, 2H), 6.82 (d, *J* = 4.8 Hz, 1H), 3.74 (s, 3H), 3.63 (s, 2H). HPLC purity 99%. MS ESI+H^+^ calc. 301.32 exp. 301.10.

### 2.2 Antiparasitic activity and cytotoxicity

The antitrypanosomatid activity of the compounds was ascertained as follows. *Leishmania* parasites were cultured at 26 °C in SDM-79 medium supplemented with 10% fetal calf serum (FCS), and the antileishmanial activity was determined according to a previously published protocol for LUC-expressing and wild-type *Leishmania* (Sereno et al., 2001, Abbassi et al., 2008). *Trypanosoma brucei gambiense* were grown at 26 °C in Cunningham's medium supplemented with 10% FCS, 2 mM GlutaMAX-1 (Gibco, USA), 100 U/ml penicillin, 100 μg/ml streptomycin and 20 mg/ml bovine hemin. The epimastigote form of *Trypanosoma cruzi* (strain Y cl7 scl2, lineage Tc2) (kindly provided by Philippe Truc and Christian Barnabé, UMR 177 IRD-CIRAD InterTryp, Montpellier, France) were grown at 26 °C in liver infusion tryptose (LIT) medium, supplemented with 10% FCS, 2 mM GlutaMAX™-I, 100 U/ml penicillin, 100 *μ*g/ml streptomycin and bovine hemin (20 mg/l) (Gibco). The antitrypanosomatids activity was determined using a protocol that was initialy set up for wild-type *Leishmania* (Abbassi et al., 2008). To assess the drug activity on *Toxoplasma gondii*, confluent human foreskin fibroblast (HFF) monolayers were infected with YFP-type I Rh kindly provided by B. Striepen, see (Striepen et al., 1998) or Tomato-type II Pru kindly provided by N. Blanchard (Toulouse), expressing parasites at 5 × 10^4^ per well, which were then centrifuged for 30 s at 1300 rpm and incubated for 30 min in a water bath at 37 °C to allow invasion. The wells were then washed three times with PBS to eliminate extracellular parasites, and the drugs were added at various concentrations ranging from 1 to 50 μM. After incubation for 24 h at 37 °C in a humidified atmosphere containing 5% CO_2_, the cells were fixed in 2.5% methanol-free formaldehyde (Tebu-bio)/PBS for 30 min at room temperature. Nuclei were stained with Hoechst 33258 (2 μg/ml, Molecular Probes) for 20 min and then washed three times with water for 10 min each time. The number of infected cells, i.e., cells harboring a parasitophorous vacuole, and the number of parasites per vacuole were determined using an Olympus ScanR microscope ( × 20 objective) and ScanR software. The parasitic index in % compared to the control was calculated as follows: (PI=((number of parasite/100 cells in treated wells) x (% of infected macrophage in treated well)/(number of parasite/100 cells in untreated wells) x (% of infected macrophage in untreated wells))*100). Then, the 50% inhibitory concentration (IC_50_) was calculated using Prism software (Prism 4 for Mac OS X, version 5.0b, December, 2008).

The human monocytic cell line THP-1 was cultivated in RPMI medium supplemented with 10% FCS, 2 mM GlutaMAX, 100 U/ml penicillin, and 100 μg/ml streptomycin. HFF cells were cultivated in Dulbecco's modified Eagle's medium (DMEM) with Earle's salts containing 10% FCS, 10 mM HEPES, 1 mM sodium pyruvate, 2 mM GlutaMAX, 100 U/ml penicillin, and 100 μg/ml streptomycin. The THP-1 and HFF cells were maintained in a 5% CO_2_ incubator at 37 °C. Cytotoxicity was evaluated via the MTT assay, and the IC_50_ values were calculated using Prism software (Prism 4 for Mac OS X, version 5.0b, December, 2008).

### 2.3 HDAC assays

The HDACi effect against HeLa nuclear extracts and recombinant proteins were measured using a fluorometric HDAC assay kit (Active Motif, Belgium) according to the manufacturer's instructions. Briefly, 30 μL of HeLa nuclear extract (Active Motif, Belgium) or various HDAC recombinant proteins (Active Motif, Belgium) was mixed with 5 μL of a 10 × compound and 10 μL of assay buffer. A negative control containing trichostatin A at a final concentration of 2 μM was analyzed for each independent experiment. A fluorogenic substrate (10 μL) was added, and the reaction was allowed to proceed for 30 min at room temperature and then stopped by the addition of a developer containing trichostatin A. The fluorescence was monitored after 30 min at excitation and emission wavelengths of 360 and 460 nm, respectively.

HDAC activity was determined from total protein extracted from *T. gondii*. Confluent cell cultures (T-175 cm^2^) were infected with 5 × 10^7^ tachyzoïtes in DMEM supplemented with 1% (v/v) FCS. Tachyzoïtes were harvested after 72 h, resuspended in RIPA buffer (0.1 M PBS, pH 7.2, 0.5% deoxycholate, 0.1% SDS, and 0.5% Nonidet P-40) and incubated for 30 min at 4 °C. After centrifugation at 13000 rpm for 30 min at 4 °C, the supernatant was collected, adjusted to a total protein concentration of 312 μg/ml and then used for determining the HDAC activity as described above.

### 2.4 Comparative modeling and ligand docking

Protein sequences were recovered from the appropriate databases for two toxoplasmal strains GT1 and Me49 as representatives of type I and type II *T. gondii* (http://toxodb.org/) and from UNIPROT for the leishmanial and human enzymes. Unfortunately the leishmanial enzymes provided poor sequence-structure alignment precluding further analysis. Meanwhile, the sequences from both strains, GT1 and ME49 revealed similar trends, so the docking study was performed on three GT1 sequences only. The best results were obtained for three sequences from *T. gondii* (A0A125YPH2, S7UUG9 and S7W8W1, *T. gondii* GT1) but not for two other sequences (S7UW66 and S7UZC5). The latter showed only partial alignment and significant sequence divergence with known crystal structures of HDACs. For those two sequences, the truncated structural alignment did not allow complete modeling of the active site, which prevented further ligand docking studies. Otherwise, for the three other sequences, active site boundaries in each structural model was deduced as the vicinity of the co-crystallized ligand (or hydroxamate compounds transferred from related HDACs by protein-protein superposition) using @TOME-2 comparative option. The same chemical entities served, in addition, to define a shape restraint to guide the docking, in absence of the zinc atom, in the automatically computed models.

All the results for the HDACs from *T. gondii* (GT1 and Me49 strains) as well as for *Leishmania infantum* and human HDACs, can be found on the following web pages (the links therein):

http://atome3.cbs.cnrs.fr/AT23/DB/HDAC_TOXGG.html
http://atome3.cbs.cnrs.fr/AT23/DB/HDAC_TOXGM.html
http://atome3.cbs.cnrs.fr/AT23/DB/HDAC_LEIIN.html
http://atome3.cbs.cnrs.fr/AT23/DB/HDAC_HUMAN.html

## 3 Results and discussion

The synthetic routes of aminophenylhydroxamate and aminobenzylhydroxamate derivatives are summarized in Fig. 1. Reaction of amine and acid chloride or sulfonyl chloride in the presence of trimethylamine afforded the amide or sulfonamide intermediates, which subsequently reacted with lithium hydroxide to obtain corresponding acids intermediates, then the acid are activated by reaction with ethyl chloroformate in the presence of N-methyl-morpholine, the intermediates react with hydroxylamine to afforded corresponding target compounds 345, 349–351, 360–363.

The compound ST3, a benzohydroxamate, exert antileishmanial activity against promastigote forms of *L. infantum* and *L. braziliensis* in the range of 20 μM but is less potent against *L. tropica* (Table 1). In addition, this compound is also active against *T. brucei* but do not display anti-*T. cruzi* activity (IC_50_ > 100 μM). It is moderately cytotoxic for THP-1 cell line or for HFF, with a determined cytotoxicity above 250 μM (Table 1). Nevertheless when tested against the intracellular form of *Leishmania* it discloses a low antiparasitic activity (IC_50_ of 140 μM and 111 μM against *L. braziliensis* and *L. infantum* respectively). As the calculated index of selectivity for ST3 is of about 5.4, this small compound (MW: 138) was considered as an interesting lead from which a series of derivative targeting trypanosomatid parasites was developed. Unexpectedly, almost all the aminophenylhydroxamates and aminobenzylhydroxamates synthetized exert low or no antitrypanosomatid activity (Table 1), irrespective of the parasite tested (*T. brucei* or *Leishmania* spp.). The sole exception is the compound named 345, which exhibited moderate antileishmanial activity in the range of 15–50 μM, *L. braziliensis* being the most susceptible *Leishmania* species. Nevertheless, this compound is highly cytotoxic to THP-1 and HFF cells.

**Table 1:** Structure of HDACi and *in vitro* activity of aminophenylhydroxamates and aminobenzylhydroxamates derivatives.

Name/cLogP/structure			Antiparasitic activity					Cytoxicity and Selectivity				HDACi	
Compound	cLogP	Structure	*L. tropica* MHOM/BR/73/5-ASKM IC_50_ μM	*L. braziliensis*^a^ MHOM/BR/75/M209 IC_50_ μM	*L. infantum*^a^ MHOM/MA/67/ITMAP-264 IC_50_ μM	*T. brucei* MHOM/CI/81/DAL967 IC_50_ μM	*T. gondi* *RH-YFP* IC_50_ μM	HFF IC_50_ μM	IS^a^	THP-1 IC_50_ μM	IS^b^	HeLa IC_50_ μM	Fold
SAHA	1.0		ND	ND	ND	ND	ND	ND	ND	ND	ND	2.100 ± 0.2	1
ST3	0.855		174.1 ± 4.5	19.6 ± 1.5 **140** ± **10.5**^b^	29.3 ± 14.4 **111.0** ± **15.3**^b^	40,7 ± 2.6	>>50	>400	ND	271.0+/_30.0	5.4	7.865 ± 0.908	0.26
345	1.545		35.3 ± 12.2	23.5 ± 1.5	27.2 ± 2.5	>60	5.0 ± 1.0	58.3 ± 1.2	11.6	1.7. ±0.5	0.3	0.866 ± 0.400	2.42
349	1.844		>60.0	>60	>60.0	>60	>>50	>400	ND	264 ± 8.0	ND	0.633 ± 0.041	3.31
350	1.420		>60.0	>60	>60.0	>60	40.0+/10.0	300.0 ± 50.0	7.5	>400	ND	8.611 ± 1.540	0.24
351	1.719		>60.0	>60	>60.0	>60	>>50	116.5 ± 3	ND	33.5 ± 15.0	<1	0.881 ± 0.083	2.38
360	1.724		55.0 ± 15.0	>60	>60.0	>60	4.9 ± 0.15	22.6 ± 9.5	4.5	2.2 ± 0.5	<1	0.399 ± 0.137	5,29
361	2.022		>60.0	>60	>60.0	>60	>>50	25.0 ± 4.0	ND	>400	ND	1.825^a^	1.15
362	1.438		>60.0	>60	>60.0	>60	52.5 ± 10.4	>400	>7.6	30.5 ± 3.0	<1	0.518 ± 0.146	4.05
363	1.829		>60.0	>60	>60.0	>60	0.35 ± 0.05 **2.27** ± **0.05**^c^	105 ± 10.5	300 **46#**	3.6 ± 0.5	<1	0.085 ± 0.035	24.70

IS: Selectivity indexes For *T. gondi* (IS^a^) and *L. infantum* (IS^b^) are calculated according to the formula: IT = IC_50_ (HFF or THP-1)/IC_50_*T. gondi* or *Leishmania infantum*.

ND: Not determined.

a Luciferase expressing strains (Sereno et al., 2001).

b Intracellular forms of Leishmania.

c Pru Type II strain of *T. gondii*.

We further evaluated the capacity of ST3 and all aminopheylhydroxamate and aminobenzylhydroxamates synthetized to act against *Toxoplasma gondii*, an intracellular parasite belonging to Phyllum Apicomplexa. For *T. gondii*, the IC_50_ of ST3 is above 50 μM. The 8 compounds synthesized express IC_50_ values ranging from more than 60 μM to less than 1 μM (Table 1). Of the 8 derivatives synthesized, 3 strongly inhibited the proliferation of a type I *T. gondii* strain at concentrations below 10 μM. These 3 compounds were 345, 360 and 363, with IC_50_ values of 5.0 μM, 4.0 μM, and 0.35 μM, respectively (Table 1). The capacity of 363 to inhibit a type II strain of *T. gondii* was found to be of 2.25 μM (see Table 1#). Among all the aminophenylhydroxamates and aminobenzylhydroxamates synthetized, the compound 363 exhibited a high selectivity for the intracellular proliferative stage of *T. gondii* (Table 1), with calculated selectivity indexes of 300 against HFF and of 10 for THP-1 cells.

**Fig. 2:**
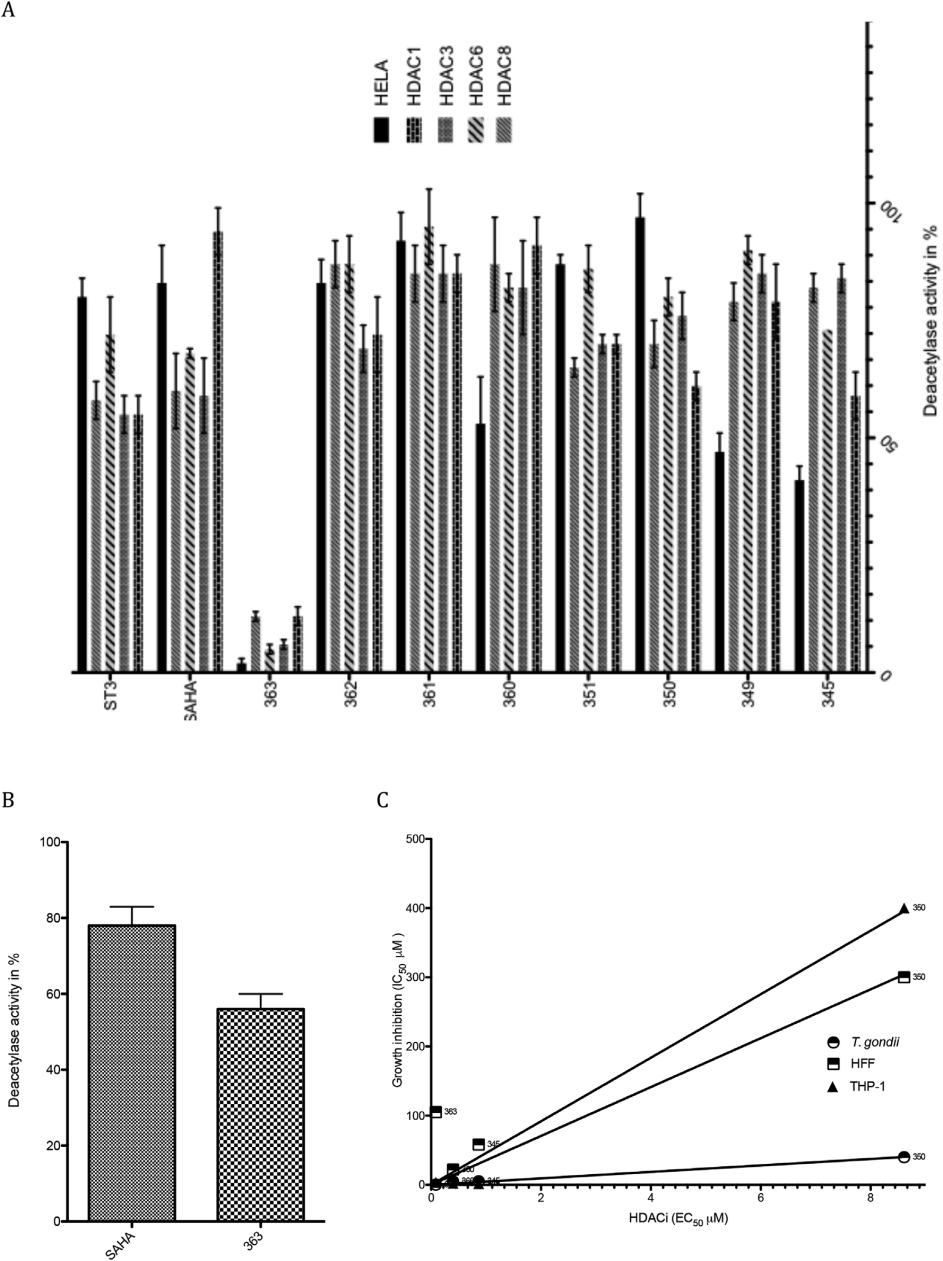
**HDACi screen and antiparasitic and cytotoxicity activities correlation. Screen for anti-HDAC activity (A)**. HDAC activity of HeLa nuclear extracts and of recombinant HDAC 1, 3, 6, and 8 proteins was a certained in the presence of 1.0 μM of each of the 8 synthesized compounds, as well as of SAHA. The experiments were carried out in triplicate, and the results are expressed as the percentage of deacetylase activity compared to the untreated control. **Inhibition of *T. gondii* deacetylase activity by SAHA and the compound 363 (B).** The total protein extract of *T. gondii* tachyzoites were prepared as described in the Materials and Methods section. The deacetylase activity was determined after the addition of 0.125 μM of compound 363 or of SAHA. The results are expressed as the percentage of deacetylase activity compared to untreated *T. gondii* protein extracts. The results are the mean of 4 experiments, carried out in triplicate. **Correlation between HDACi and anti *T. gondii* activity or cytotoxicity potency (C).**

In a HeLa cell nuclear extract, HDAC 1, 2, 6, and 8 are the predominant HDACs. To compare our results with previously reported data, we used a well-known HDACi, vorinostat (SAHA). We obtained an IC_50_ for vorinostat in the micromolar range (2 μM) (see Table 1), which agrees with the literature data on this molecule (1.3 μM) (Jiao et al., 2009). For all the other compounds, the determined IC_50_ ranges from more than 1.8 μM for the less active compounds (ST3, 350 and 361) to 0.085 μM for the more active compound (363). This latter inhibitory concentration is lower than the IC_50_ that we determined for SAHA and more than 20-fold lower than those of well-known HDACis. To further characterize the HDACi effect of our series of compounds, we first ascertained the inhibitory effect of all the compounds at a single concentration of 1.0 μM against HeLa nuclear extract and recombinant HDAC1, 3, 6, and 8. As shown in Fig. 2A, the sole compound that exhibited a strong HDACi effect at 1.0 μM is compound 363 (90% inhibition) (Fig. 2A). We further investigated the specificity of 363 against a set of recombinant HDAC enzymes (HDAC1, 3, 6, and 8) (Table 2) and found that 363 is more potent for HDAC6 (Class II HDAC) as compared to the other HDACs, that belong to the class I. We then assessed the effect of 363 on *T. gondii* HDAC activity. As shown in Fig. 2B, at 0.125 μM, compound 363 exerted a strong inhibitory effect on the deacetylase activity measured in the total protein extract of *T. gondii*. Compound 363 inhibited 44% of the *T. gondii* deacetylase activity, whereas SAHA inhibited only 22% of this activity (Fig. 2B). The compound 363 is structurally related to the HDAC6 inhibitor Nexturastat or BRD73954 (Wagner et al., 2013, De Vreese and D'hooghe, 2017) and it will be interesting to ascertain Nexutarastat's anti *T. gondii* pontency in the future.

**Table 2:** HDAC inhibitory effect of the compound 363 as compared to SAHA.

rHDAC	363 IC_50_ μM	SAHA IC_50_ μM	Fold
HDAC 1	0.257 ± 0.050	0.627 ± 0.039	2.43
HDAC 3	0.160 ± 0.052	0.170 ± 0.014	1.06
HDAC 6	0.045 ± 0.015	1.573 ± 0.800	34.9
HDAC 8	0.863 ± 0.045	0.230 ± 0.130	0.26

Among the 8 compounds, those with the strongest anti-*Toxoplasma* activity, *i.e IC*_*50*_ < 50 μM (345, 350, 360, 363), are among the best HDACis of the series. A high correlation between HDACi and *T. gondii* activity is recorded (R^2^ = 0.9950). In addition, their cytotoxic activity against HFF and THP-1 correlates also well with their HDACi (R^2^ = 0.9000 and R^2^ = 0.9933) respectively (Fig. 2C). But the correlations appear not to be obvious when compounds with low anti *T. gondii* activity IC_50_ > 50 μM (ST3, 349, 351, 352) were included in the analysis, R^2^ = 0.3758, 0.4437 and 0.6667 for *T. gondii*, HFF and THP-1 respectively (see Supplementary data S2). Therefore, improved anti-*T. gondii* potency of the series is largely dependent on potent HDACi activity. However, since three compounds exhibit improved HDACi activity that does not lead to improved anti-*T. gondii* activity it is likely that other factors are also involved in the modulation of their cytotoxicity and anti *T. gondii* potency. Among them, the capacity of each compound to reach their intracellular targets and/or to readily inhibit HDAC activity might play a role. The THP-1 cell line, which was derived from the peripheral blood of a 1-year-old human male with acute monocytic leukemia, is known to be highly susceptible towards hydroxamate derivatives (Sung et al., 2010), and HDACis are known to exhibit relatively selective cytotoxicity against growing malignant cells compared to healthy ones (Popovic and Licht, 2012). Non-malignant cells such as HFFs are less susceptible to the synthesized compounds. Nevertheless, to gather more information on the selectivity of 363, we investigated its cytotoxicity in the HEPG2 cell line, which is considered a model system for studies on liver metabolism and xenobiotic toxicity. Compound 363 is mildly cytotoxic for HEPG2 cells, with an IC_50_ of greater than 15 μM. The calculated index of selectivity of 42 confirms the high selectivity of this compound for *T. gondii* tachyzoites.

**Fig. 3:**
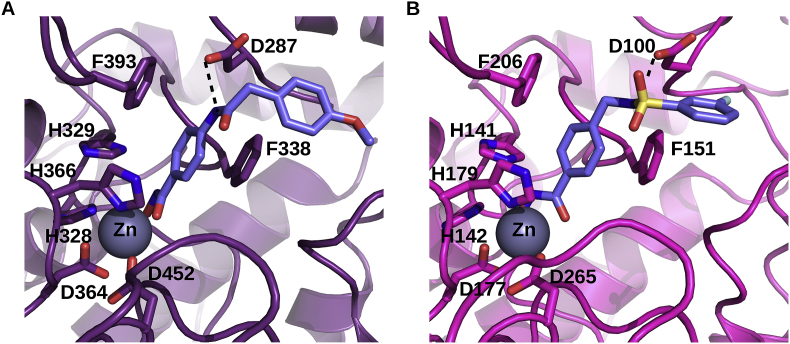
**Docking of two inhibitors in the models of two TgHDACs. Model of S7W8W1 from *T. gondii* GT1 in complex with ligand 363 (A)**. **Model of A0A125YPH2 from *T. gondii* GT1 in complex with ligand 350 (B).** Both models were deduced automatically using the server @TOME-2 and ligands were docked using PLANTS and a shape restraint extracted from a co-crystallized ligand. The putative binding site of the zinc atom is indicated by the symbol Zn. The chelating sidechains are shown as wireframe. Similarly the two phenylalanine and the aspartate residues interacting with the benzohydroxamate inhibitors are shown (discussed in the text). Only one hydrogen bond (black dashed line) is predicted and shown for each compound beside the interactions involving the hydroxamate group which are not shown for clarity. The zinc atom is shown as a grey sphere. The figure was drawn using pymol (www.pymol.org).

The currently identified HDACi-based anti-*T. gondii* compounds target class I HDACs. Among the 5 HDAC-encoding genes in the genome of the GT1 strain of *T. gondii* (www.toxodb.org/), 3 are class I HDACs, one is a class IV HDAC, and 1 is a class II HDAC. Fold recognition using the server @TOME-2 (Pons and Labesse, 2009) was used to yield sequence-structure alignments with well-characterized HDACs and to derive models of the parasite enzymes. First, it highlighted strong similarities for two *Toxoplasma gondii* TgHDACs with *Homo sapiens* HsHDAC2 (with 56% and 64% of sequence identity over ∼360 residues). Two other TgHDACs showed weaker similarities with bacterial HDACs (40% identity), being far more distant to the *Homo sapiens* HsHDADC6 (∼20% identity). The last one appeared to encode only a partial HDAC fold and might correspond to a pseudogene. Taking advantage of the comparative docking implemented in @TOME-2, active site delimitations and pharmacophoric shape restraints could be defined to allow focused screening performed using PLANTS (Korb et al., 2007). The docking of the lead compound ST3 was rather straightforward. It perfectly matches the binding mode of the phenyl-hydroxamate moiety of Nexturastat as observed in HDAC6 from *Danio rerio* (PDB5G0J) (Miyake et al., 2016). Satisfactory docking poses for most the other benzohydroxamates were also obtained for the three targets we could model. In all cases, the common aromatic phenyl moiety showed favorable interactions with two aromatic side-chains (mainly phenylalanines) lying at the entrance of the active site (Fig. 3). A neighboring aspartate which is also conserved at the edge of the active site is pointing toward the amide/sulfonamide groups present in the chemical series derived from ST3, and accordingly it may (or may not) form a favorable hydrogen bond with the incoming ligand. This could explained the observed change in affinity depending on the position of the amide/sulfonamide relative to the phenyl-hydroxamate moiety. In the case of the leishmanial HDACs, significantly lower similarities were detected (25–40% sequence identity) with more important insertion/deletions. This more pronounced divergence precluded proper modeling of the active site entrance and ligand docking. But this divergence could potentially explain the detrimental impact of the substitutions we designed on our lead compound. All these targets deserve further characterization to guide the design of more potent compounds.

For the first time, we have demonstrated that a potent inhibitor of the mammalian class IIb of HDACs (HDAC6) is effective as an anti-*T. gondii* compound. The molecular target now needs to be further characterized in *T. gondii*. Unexpectedly, among the 8 derivatives synthesized there was minimal efficacy against Trypanasomes and only one compound with demonstrated antileishmanial activity (345), but this compound also exhibited high *in vitro* cytotoxicity. In order to address whether these HDAC inhibitors can be developed to target intracellular parasites more broadly, we will synthesize new compounds from the ST3 and or the 345 molecules in order to improve their efficacy against Leishmania parasites and decrease their cytotoxicity.

*T. gondii* exists in two life stages, the rapidly proliferating tachyzoïte form and the latent encysted bradyzoïte form, the latter of which remains in the body for the lifetime of the host, maintaining the risk of recurrence. There are currently no effective treatments against the bradyzoïte form (Neville et al., 2015), and the medicines that target the tachyzoïte form (pyrimethamine and sulfadiazine) are associated with toxicity and hypersensitivity (Butler et al., 2013). Currently characterized HDACis with anti-*T. gondii* activity (Strobl et al., 2007, Maubon et al., 2010, Bougdour et al., 2009) require high concentrations to inhibit parasite growth and show little selectivity for the intracellular stage of *T. gondii*. This relatively low specificity of these HDACis limits the development of an HDACi-based *T. gondii* therapy.

Among the compounds synthesized, those expressing strong anti-*Toxoplasma* activity are among the best HDACis of the series, particularly compound 363, the most effective HDACi with high selectivity for *Toxoplasma*. The IC_50_ of 363 is equivalent, in terms of *in vitro* efficiency, to the IC_50_ pyrimethamine the classical anti-*Toxoplasma* drug (IC_50_ of 0.35 for 363 and 0.4 μM for pyrimethamine) (Butler et al., 2013). We propose that compound 363 be considered as a drug-like compound, whose capacity to act against *T. gondii* infection *in vivo* must now be probed.

## Appendix A Supplementary data

SuppDataS2 SuppDataS2

SupDataS1_7BE7 SupDataS1_7BE7
