# Supplementary figures and images for: Synthesis of aminophenylhydroxamate and aminobenzylhydroxamate derivatives and in vitro screening for antiparasitic and histone deacetylase inhibitory activity
Source: Int J Parasitol Drugs Drug Resist. 2018 Jan 31;8(1):59–66. doi: 10.1016/j.ijpddr.2018.01.002 (PMC6114082; doi:10.1016/j.ijpddr.2018.01.002)

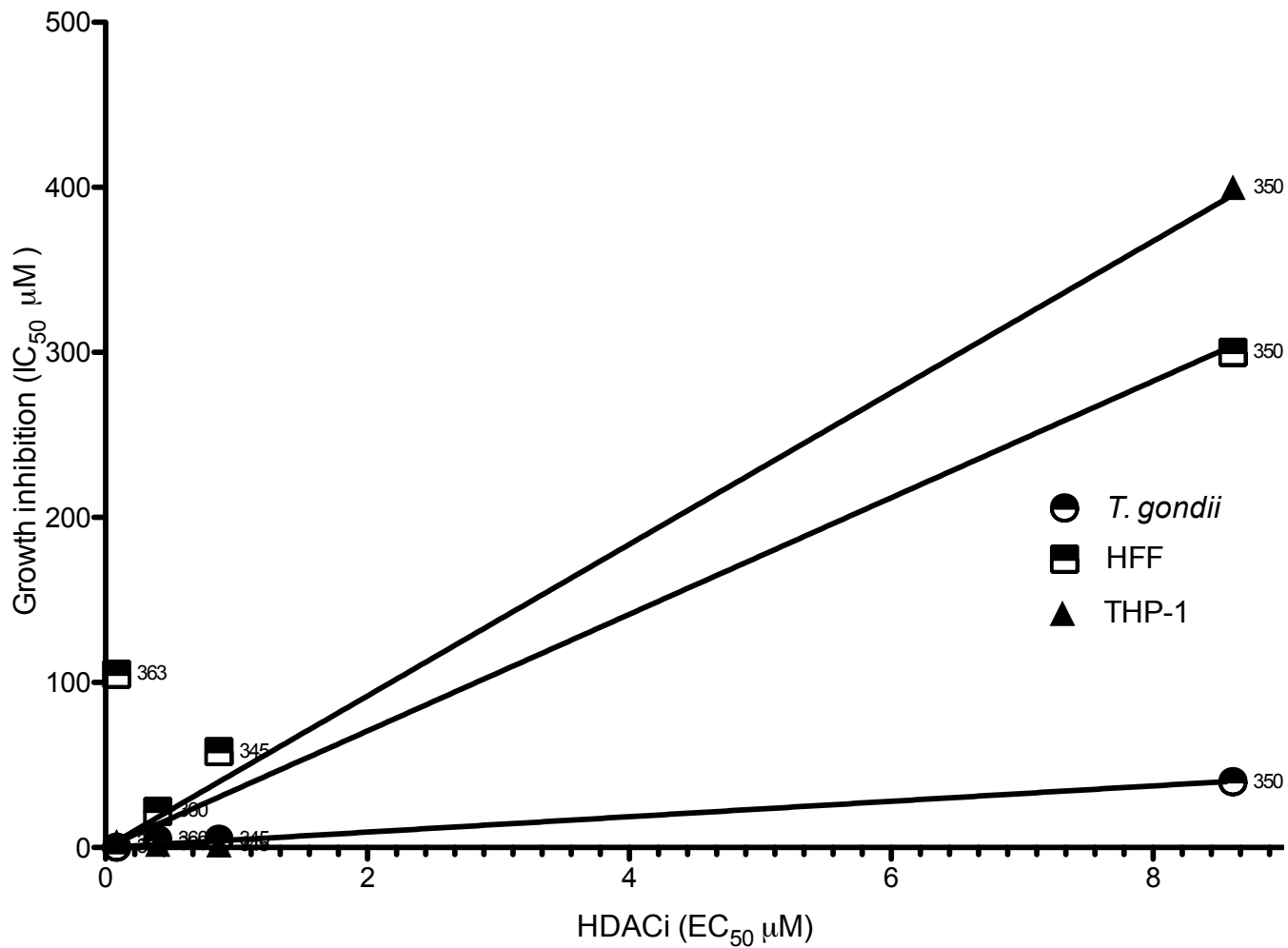

Supplement: SuppDataS2 [file mmc1.pdf]
