# Supplementary material for: Synthesis of aminophenylhydroxamate and aminobenzylhydroxamate derivatives and in vitro screening for antiparasitic and histone deacetylase inhibitory activity
Source: Int J Parasitol Drugs Drug Resist. 2018 Jan 31;8(1):59–66. doi: 10.1016/j.ijpddr.2018.01.002 (PMC6114082; doi:10.1016/j.ijpddr.2018.01.002)
Supplement: SupDataS1_7BE7 [file mmc2.docx]

Supplementary Figure 1. ^1^H NMR (200 MHz, DMSO-*d6*) spectrum of **345**

Supplementary Figure 2. ^1^H NMR (200 MHz, DMSO-*d6*) spectrum of **349**

Supplementary Figure 3. ^1^H NMR (200 MHz, DMSO-*d6*) spectrum of **350**

Supplementary Figure 4. ^1^H NMR (200 MHz, DMSO-*d6*) spectrum of **351**

Supplementary Figure 5. ^1^H NMR (200 MHz, DMSO-*d6*) spectrum of **360**

Supplementary Figure 6. ^1^H NMR (200 MHz, DMSO-*d6*) spectrum of **361**

Supplementary Figure 7. ^1^H NMR (200 MHz, DMSO-*d6*) spectrum of **362**

Supplementary Figure 8. ^1^H NMR (200 MHz, DMSO-*d6*) spectrum of **363**
